# Physiological adaptations to weight loss and factors favouring weight regain

**DOI:** 10.1038/ijo.2015.59

**Published:** 2015-05-26

**Authors:** F L Greenway

**Affiliations:** 1 Pennington Biomedical Research Center, Louisiana State University System, Baton Rouge, LA, USA

## Abstract

Obesity is a major global health problem and predisposes individuals to several comorbidities that can affect life expectancy. Interventions based on lifestyle modification (for example, improved diet and exercise) are integral components in the management of obesity. However, although weight loss can be achieved through dietary restriction and/or increased physical activity, over the long term many individuals regain weight. The aim of this article is to review the research into the processes and mechanisms that underpin weight regain after weight loss and comment on future strategies to address them. Maintenance of body weight is regulated by the interaction of a number of processes, encompassing homoeostatic, environmental and behavioural factors. In homoeostatic regulation, the hypothalamus has a central role in integrating signals regarding food intake, energy balance and body weight, while an ‘obesogenic' environment and behavioural patterns exert effects on the amount and type of food intake and physical activity. The roles of other environmental factors are also now being considered, including sleep debt and iatrogenic effects of medications, many of which warrant further investigation. Unfortunately, physiological adaptations to weight loss favour weight regain. These changes include perturbations in the levels of circulating appetite-related hormones and energy homoeostasis, in addition to alterations in nutrient metabolism and subjective appetite. To maintain weight loss, individuals must adhere to behaviours that counteract physiological adaptations and other factors favouring weight regain. It is difficult to overcome physiology with behaviour. Weight loss medications and surgery change the physiology of body weight regulation and are the best chance for long-term success. An increased understanding of the physiology of weight loss and regain will underpin the development of future strategies to support overweight and obese individuals in their efforts to achieve and maintain weight loss.

## Introduction

Obesity is a major global health problem, with 500 million obese individuals worldwide.^1^ In the United States (US) alone it was reported that 35.7% of the adult population (78 million) and 16.9% of children and adolescents (12.5 million) were regarded as obese in the period 2009–2010,^2^ with the proportion of overweight and obese individuals plateauing in the US,^3^ but continuing to rise around the world.^4^ Obesity is a significant health concern because it predisposes individuals to several comorbidities, including hypertension, dyslipidaemia, coronary heart disease, type 2 diabetes, stroke, cancer and osteoarthritis^2, 5, 6^ and a shortened life expectancy while impairing the quality of life.^7, 8^

Weight gain is the result of an imbalance between total energy intake and total energy expenditure (TEE),^5, 9^ and it is thought that substantial and sustained increases in total energy intake in the past three decades have led to the increase in body weight across the global population.^9^ Consequently, it appears that obesity is the result of flawed food intake behaviour combined with an imbalance in energy uptake and expenditure that can be rectified by caloric restriction and increased physical activity.^10^ Indeed, interventions based on lifestyle modification (for example, improved diet and exercise) are integral components in the management of obesity.^5^ However, although weight loss can be achieved through dietary restriction and/or increased physical activity, over the long term, many individuals regain the weight they have lost.^11^ The proportion of individuals who successfully maintain weight loss varies according to how ‘successful maintenance of weight loss' is defined. Wing and Hill^12^ proposed the following definition: ‘intentionally losing⩾10% of initial weight and keeping it off for⩾1 year' based on this definition, approximately a quarter of overweight individuals report successfully maintaining weight loss.

Given that the effects of diet and exercise interventions alone do not seem sufficient to support the long-term maintenance of a reduced weight, it is apparent that the problem is more complex and that obesity could be regarded as a neurobiological disease with a psychological element.^10^ There has been much research into the processes and mechanisms that underpin weight regain after weight loss. The aim of this review is to provide an overview of this research and its implications for clinical practice.

## Methods

An electronic literature search was performed using the PubMed database for relevant articles published between 01 January 2008 and 30 April 2014. Given the broad nature of this review, a structured, rather than systematic, search strategy was conducted to identify relevant articles. An initial search, restricted to English-language articles and using the following key search terms: (obesity/obese/overweight) 'and' (weight) 'and' (gain/regain*) 'and' (loss/reduc*/decreas*) identified a large number (4314) of potential articles. Therefore, additional searches were then conducted to identify articles that focused on specific aspects related to this review, using the key search terms in conjunction with the following additional topics and terms: ‘physiologic*/biologic*' ‘adapt/adaptive/adaptation/homoeostasis/homoeostatic/maintain/maintaining/maintenance' ‘metabolic/metabolism' ‘energy/energetic' ‘central/peripheral' ‘hormone/hormonal' ‘ghrelin' ‘leptin' ‘insulin' ‘pancreatic polypeptide/PP' ‘peptide YY/PYY' ‘cholecystokinin/CCK' ‘amylin' ‘glucagon-like peptide-1/GLP-1' ‘psycholog*/neuropsycholog*' ‘food intake' ‘appetite' ‘exercise/physical activity' ‘genetic' and ‘hedonic'. Following searches, titles and abstracts of articles were scanned to determine their relevance to the scope of this review. Articles were included if they were deemed to provide relevant information related to the scope of physiological adaptations to weight loss and factors that favour weight regain. References from bibliographies of selected articles, including reviews, original research articles and other articles of interest were scanned for additional relevant supporting articles, and data quality was determined by publication in peer-reviewed literature. Selection of articles was also based on the author's own judgement, clinical experience, perspective and knowledge of the literature, as well as additional searches that were performed in order to address journal peer-reviewers' comments. Articles that were not deemed pertinent to the topics covered in the review, as well as single case studies, short commentaries, letters and interviews were excluded. Overall, a total of 106 articles were included in the review.

### Processes involved in the regulation of body weight

**Figure 1:**
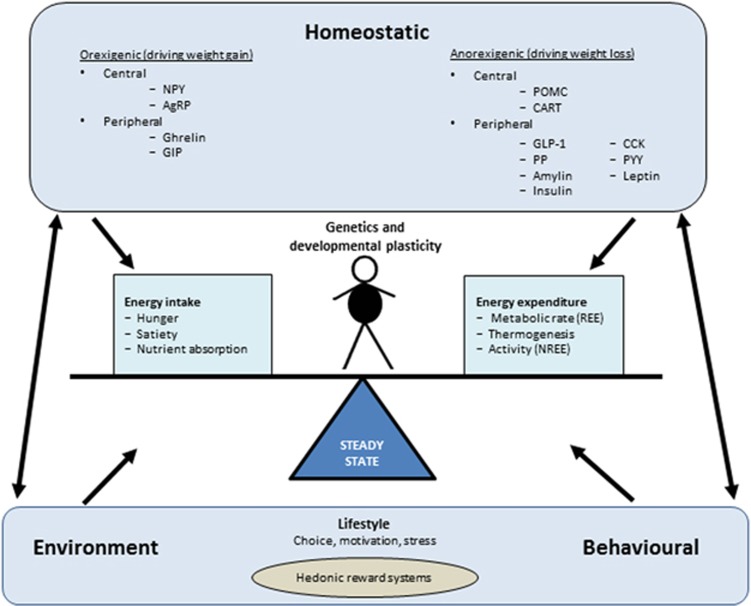
Factors affecting energy balance and thus steady-state weight. There are three main groups of factors—homoeostatic, environmental and behavioural processes—that interact and influence steady-state body weight. Alterations in any of these factors will result in changes to this steady-state and could result in obesity. AgRP, agout-related peptide; GIP, gastric inhibitory polypeptide; GLP-1, glucagon-like peptide-1; CART, cocaine- and amphetamine-regulated transcript; CCK, cholecystokinin; PYY, peptide YY; NPY, neuropeptide Y; POMC, pro-opiomelanocortin; PP, pancreatic polypeptide; REE, resting energy expenditure; NREE, non-resting energy expenditure. ‘Central' and ‘peripheral' refer to the site where the molecules are produced, rather than where they necessarily act. In gthe brain, insulin acts as an anorexigenic hormone.^13, 104, 105^ However, in the periphery, insulin lowers blood sugar, which potently stimulates food intake.^106^

Under steady-state conditions energy intake (food) is metabolised and used to fuel basal metabolism, thermogenesis and our energy expenditure (physical activity).^13^ Any excess is stored as fat in adipose cells for later use. There is a genetic contribution to the determination of an individual's weight with early-life events and parental guidance also playing a part,^13, 14, 15^ but ultimately steady-state body weight is influenced by a number of different factors. These factors fall into three distinct but interrelated categories: homoeostatic, environmental and behavioural processes (Figure 1).^11^

#### Homoeostatic processes

Body weight is regulated by a complex neuro-hormonal system,^11, 16^ which reflects the fundamental biological importance of energy balance and nutrient supply.^13^ A full overview of this biological system is beyond the scope of this article and has been reviewed in detail elsewhere.^13^ In essence, signals involved in the homoeostatic regulation of food intake, energy balance and body weight are integrated centrally in the arcuate nucleus of the hypothalamus,^17^ the caudal brainstem and parts of the cortex and limbic system.^13^ A number of neuropeptides and hormones involved in appetite regulation function centrally in the hypothalamus; some (for example, neuropeptide Y (NPY) and agouti-related peptide (AgRP)) are orexigenic (stimulate hunger), while others (for example, pro-opiomelanocortin (POMC) and cocaine- and amphetamine-regulated transcript) are anorexigenic (suppress hunger).^13, 18^ The hypothalamus also processes peripheral signals that convey information about short-term food intake (that is, nutrient availability) or long-term energy balance (that is, energy stores) to achieve energy homoeostasis.^11, 19^ A feedback loop is created between the brain and periphery (gastrointestinal tract, pancreas, liver, muscle and adipose tissue).^11, 13^ Short-term signals include the orexigenic hormones ghrelin and gastric inhibitory polypeptide; the anorexigenic hormones glucagon-like peptide-1 (GLP-1), peptide YY (PYY) and cholecystokinin (CCK) from the gastrointestinal tract; the anorexigenic hormones pancreatic polypeptide (PP), amylin and insulin from the pancreas; and the anorexigenic hormone leptin from adipocytes.^13, 17, 19^ Insulin, however, is unique, since it reduces food intake centrally, but causes weight gain when used peripherally to treat diabetes. The hypothalamus also integrates signals from ‘hedonic' reward pathways in the cortico-limbic system, associated with the palatability (for example, sight, smell and taste) of food.^13^ Such hedonic reward pathways can override the homoeostatic system and increase desire to consume energy-rich food, despite physiologic satiation and replete energy stores.^13, 20^ Several neurotransmitter systems in the brain, including the dopaminergic, opioidergic and cannabinoid mechanisms, have a major role in reward pathways and mediating the pleasure drive for eating.^20, 21, 22^

It has been suggested that, rather than something being ‘wrong' with homoeostatic control of food intake, the system is insufficiently powered to cope with radical environmental changes and, thus, overwhelmed to the point where activation of the hedonic pathways becomes a major driving force for overconsumption.^23^ Recently, evidence has emerged proposing an additional mechanism by which homoeostatic control of food intake can be overridden. Dietary intake of saturated fatty acids induces inflammation in the hypothalamus, a process mediated by glial cells, which may lead to changes in neuronal function and result in disturbances to leptin responsiveness and food intake.^24, 25, 26, 27^ Glial cells may, therefore, have an important role in the regulation of body weight, with chronic activation of glial cells linked with the perpetuation of obesity and the onset of related complications.^25, 26, 28, 29^

#### Environmental

The environment in which we live has an important role in influencing energy homoeostasis. Current levels of obesity are attributable, at least in part, to an ‘obesogenic' environment that impacts cortico-limbic brain areas concerned with learning and memory, reward, mood and emotion.^30^ Contributing factors to this environment include intense marketing of energy-dense foods, increased availability of these foods and increased portion sizes, which all present people with the opportunity to over-consume large portions of sugary and high-fat foods.^31, 32^ Moreover, a high-stress society stimulates compensatory food intake.^15^ This increase in food intake is coupled with decreases in physical activity, for example, because of sedentary jobs^15^ and a decline in the promotion of physical education in schools.^31, 32^ Ultimately, an ‘obesogenic' environment makes it more challenging for individuals to maintain a healthy body mass index (BMI) through diet restriction or maintaining healthy levels of physical activity.

A number of other factors have also been postulated by McAllister *et al.*^32^ to contribute to obesity, including infection, epigenetics, increasing maternal age, greater fecundity among people with higher adiposity, assortative mating, sleep debt, endocrine disruptors, pharmaceutical iatrogenesis, reduction in variability of ambient temperatures, through to intrauterine and intergenerational effects (Table 1). Our understanding of the contribution of these factors to obesity is variable, with much evidence based on epidemiological and pre-clinical data,^32^ but there is increasing interest in the literature and a number of factors are worthy of further consideration. As McAllister *et al.* note, with an increasing prevalence over time of many of these proposed factors (for example, sleep debt and epigenetics) combined with an increasing prevalence of obesity, further research into the impact of these factors in modulating obesity is warranted (Table 1).

#### Behavioural

How we behave also influences our energy homoeostasis. It is apparent that simply knowing that a healthy diet and exercise will result in weight loss is not sufficient to reach and maintain a healthy lifestyle and reduce excess body weight.^15^ Behaviour patterns are a fundamental contributor to the aetiology of obesity and, therefore, behavioural therapy is often a key part of the management of obese individuals.^33^ A proportion of obese individuals do successfully maintain weight loss and this is associated with specific changes in behaviour, particularly with regard to diet and exercise.^33^ In a recent study, 110 obese women who completed a 6-month lifestyle intervention were assessed in terms of weight loss maintenance over a 3.5-year period.^34^ Those candidates who maintained weight loss (⩾5% reduction in body weight) exhibited more frequent self-monitoring of food and calorie intake, selected lower calorie foods, planned meals in advance and weighed regularly.^34^ Recent findings from the National Weight Control Registry—comprising over 2800 individuals who have maintained a weight loss of ⩾13.6 kg for⩾1 year—demonstrate that sustained behaviour change can lead to long-term maintenance of weight loss.^35^

Personal motivation for change can have a fundamental role in modifying unhealthy habits and lifestyle.^15^ The importance of promoting self-efficacy in increasing physical activity in obese individuals has recently been highlighted; for example, improvements in self-efficacy following interventions have been shown to correlate with improved physical activity behaviour.^36^ Self-efficacy is the ‘belief that an individual has the ability to successfully engage in a specific behaviour such as exercise'.^36^ In a meta-analysis of 61 studies, four behaviour change techniques (‘action planning', ‘time management', ‘prompt self-monitoring of behavioural outcome' and ‘plan social support/social change') were significantly associated with positive changes in self-efficacy. ‘Prompt self-monitoring of behavioural outcome' and ‘plan social support/social change' and an additional 19 behaviour change techniques were also associated with positive changes in physical activity.^36^ The concept of discrepancy, the contradiction between how a person currently sees him/herself and how he/she would like to be, in order to correspond to his/her ideal self-image, value system and expectations, as well as the concept of self-regulation, are also recognised as important components in realising behaviour change.^15^

### Physiological adaptations to weight loss and factors favouring weight regain

**Figure 2:**
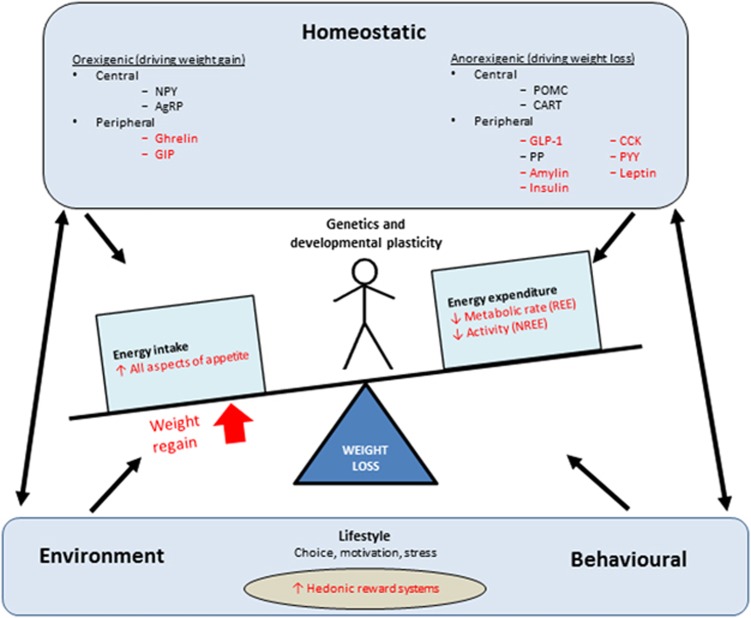
Physiological factors driving weight regain after weight loss. Changes in specific parameters that drive weight regain are indicated in red. AgRP, agout-related peptide; GIP, gastric inhibitory polypeptide; GLP-1, glucagon-like peptide-1; CART, cocaine- and amphetamine-regulated transcript; CCK, cholecystokinin; PYY, peptide YY; NPY, neuropeptide Y; POMC, pro-opiomelanocortin; PP, pancreatic polypeptide; REE, resting energy expenditure; NREE, non-resting energy expenditure. ‘Central' and ‘peripheral' refer to the site where the molecules are produced, rather than where they necessarily act. In gthe brain, insulin acts as an anorexigenic hormone.^13, 104, 105^ However, in the periphery, insulin lowers blood sugar, which potently stimulates food intake.^106^

Evidence continues to accumulate that the compensatory changes in biological pathways involved in appetite regulation, energy utilisation and storage encourage weight regain following weight loss. These changes affect our complex neuro-hormonal system that regulates energy homoeostasis, including perturbations in the levels of circulating appetite-related hormones and energy homoeostasis, as well as alterations in nutrient metabolism and subjective appetite (Figure 2).

#### Levels of circulating hormones

Appetite-related hormones have a key role in weight regain after weight loss.^37^ With the exception of increases in PP,^19, 38^ changes in hormones following weight loss tend to favour weight regain by increasing hunger and promoting energy storage.^19, 38^ For example, following diet-induced weight loss, there are increases in levels of ghrelin,^39^ and gastric inhibitory polypeptide^19^ with decreases in levels of leptin,^19, 40^ PYY,^41^ CCK,^42^ amylin,^19, 43^ insulin^19, 38, 43^ and GLP-1.^44, 45^ Findings from a study of 50 overweight or obese individuals demonstrated that such hormonal alterations in response to weight loss, following a 10-week very-low-energy diet, can persist long term (~1 year).^19^ One year after the initial weight loss, significant differences (*P*<0.05) from baseline were observed in mean levels of leptin, PYY, CCK, insulin, ghrelin, gastric inhibitory polypeptide, PP and GLP-1.^19^ Thus, these findings suggest that compensatory alterations in circulating mediators of appetite, which promote weight regain following a diet-induced weight loss, are not a transient response to weight loss.

Interestingly, findings from a study of 104 obese and/or overweight individuals showed that, after diet-induced weight reduction, those who regained⩾10% of the lost weight appeared to have consistently higher baseline (fasting) leptin levels and lower baseline (fasting) ghrelin levels 6 months later versus those that maintained body weight.^37^ Because leptin and ghrelin are satiety and orexigenic signals, respectively, it may be expected that individuals who regain lost weight would have lower levels of leptin and higher levels of ghrelin.^37^ However, the findings appear to be counterintuitive and suggest that weight regain is associated with a disruption in the sensitivity to these hormones.^37^

Following gastric bypass surgery, levels of ghrelin are extremely low,^39^ while GLP-1 and PYY are elevated,^46^ which should attenuate appetite. These findings raise the possibility that the gastric bypass procedure reduces weight, at least in part, by altering the production and/or release of these mediators of appetite. Interestingly, among individuals who underwent gastric bypass, plasma ghrelin levels did not oscillate in relation to meals and were much lower than those of normal-weight controls and matched obese controls, after substantial weight loss resulting from a 6-month dietary programme.^39^ However, recent studies in rodents have indicated that weight loss following sleeve gastrectomy is not mediated by changes in ghrelin or GLP-1, or through the melanocortin (MC)-4 receptor in the hypothalamus.^47, 48, 49^ Instead, as shown by a knockout mouse study, the mechanism of weight loss in sleeve gastrectomy appears to involve the nuclear bile acid receptor, farnesoid X receptor.^50^ Note that the observation that gut hormones such as ghrelin and GLP-1 are not involved in the mechanism of weight loss with sleeve gastrectomy does not mean that they are not important mediators of body weight. Indeed, a rationally designed monomeric peptide has been shown to reduce body weight and diabetic complications in rodents by acting as an agonist at three metabolically related peptide hormone receptors: the GLP-1, gastric inhibitory polypeptide and glucagon receptors.^51^

#### Energy balance

Energy expenditure varies according to changes in body weight, and the balance between ingested energy (in the form of calories) and basal energy demand of the body is a fundamental determinant in the control of body weight.^5^ Energy balance is an integral component of many quantitative models of body weight change, which can provide useful information about the dynamics of weight loss and regain.^23, 52^ In such models, TEE encompasses resting energy expenditure (REE, that is, energy needed to fuel cellular functions), non-REE (that is, energy expended during physical activity) and the thermic effect of feeding (that is, energy needed to process ingested food).^17^

Maintenance of a reduced body weight is associated with compensatory changes in energy expenditure, which tend to favour weight gain.^53^ Diet-induced weight loss leads to a decrease in TEE, REE and non-REE.^53, 54^ Mechanisms involved in the decline of TEE following weight loss are likely linked to a reduction of body mass and enhanced metabolic efficiency.^11^ If less total mass must be moved during physical activity, the same activity will have less energetic cost, resulting in a decrease in non-REE, if levels of physical activity are kept the same.

Decreased energy expenditure after weight loss would matter little if energy intake was proportionately reduced;^55^ however, during attempts to maintain weight loss, there can be an apparent disconnect between energy intake and output that favours weight regain. A study investigating body composition and energy expenditure in 16 severely obese (body mass index 49.4 kg m^−^^2^) individuals competing in a nationally televised 30-week weight loss programme of diet restriction and vigorous exercise, demonstrated a disproportionate slowing of REE during weight loss, despite relative preservation of fat-free mass.^56^ At 30 weeks, on average, greater than one-third of the initial body weight had been lost, comprising 83% from fat and 17% from fat-free mass. However, the REE reduced by 789 kcal d^−1^, which was 504 kcal d^−1^ greater than expected, based on the change of body weight and composition.^56^ Persistence of this metabolic adaptation during maintained weight loss could predispose individuals to weight regain.

Analysis using a computational model of metabolism showed that maintenance of weight loss among contestants participating in the television show could be achieved with a feasible sustained behaviour change, comprising an average energy intake of 3000 kcal d^−1^ and 20 min per day of vigorous activity.^57^ A slow rate of weight gain was simulated if individuals were to return to their original diet and sedentary lifestyle,^57^ consistent with findings suggesting that the body weight response to a change of energy intake is slow.^58^ Note that this study evaluated the role of both diet and exercise on weight loss.^56, 57^ A recent paper discussed the effect of exercise in isolation on weight loss in 30 overweight or obese women (body mass index 30.6 kg m^−^^2^) who completed 12 weeks of supervised aerobic exercise.^59^ The study showed that 43% of participants experienced a greater-than-expected decline in REE (–102.9±77.5 kcal per day), that is, metabolic adaptation, although notable variability existed in the adaptive metabolic response to exercise.^59^ Importantly, the study findings showed that both energy expenditure and energy intake are influenced by the adaptive metabolic response to exercise-induced weight loss.^59^

#### Nutrient metabolism

The composition of the diet used for weight loss may influence subsequent weight regain.^5^ The biological effects, including energy expenditure, of dietary composition during weight loss maintenance were investigated in a study of 21 obese or overweight adults.^54^ Among individuals who lost 10–15% body weight after receiving a low-fat, low-glycaemic index or very-low carbohydrate diet for 4 weeks, TEE and REE decreased most in the low-fat diet group and least in the very-low carbohydrate group.^54^ It was suggested that a low carbohydrate diet may help protect against weight regain.

Findings from studies in obese rats showed that weight loss from dietary energy restriction was initially accompanied by a preference for the utilization of lipids over carbohydrates.^60^ However, maintained weight loss was accompanied by a shift in fuel utilization towards carbohydrate oxidation that continued during weight regain.^60^ Rodent studies also demonstrated a suppression of dietary fat oxidation during weight regain after sustained weight reduction.^61^ Increased carbohydrate utilization would spare dietary fat from oxidation, making it available for deposition and storage in adipose tissue.^60, 61^

Studies in humans demonstrate that post-obese individuals have low rates of fat oxidation^62^ and, in particular, suppressed post-prandial fat oxidation,^63^ which may explain a propensity to regain weight following weight loss.

#### Subjective appetite

Eating stimulates brain centres involved in pleasure and reward, which helps explain why the motivation to consume food, possibly even despite a state of satiety, goes beyond the need to maintain energy homoeostasis and body weight.^20^ Overeating likely reflects an imbalance in the control exerted by the hypothalamus versus reward circuits, and/or a shift in the hedonic set point for food reward.^20^ Interestingly, a reduction in striatal dopamine D2 receptors has been demonstrated in obese individuals, which may lead to overconsumption of food as a means of compensating for decreased activation of the dopamine pathway.^64^ Moreover, results from a long-term (56 weeks), randomized Phase 3 study in 1496 obese (body mass index 30–45 kg m^−^^2^) or overweight (27–45 kg m^−^^2^ with dyslipidaemia and/or hypertension) individuals, demonstrated that treatment with the combination of naltrexone/bupropion led to improvements in patients' ability to control their eating and resist food cravings.^65^ The opioid antagonist naltrexone and atypical antidepressant bupropion, which inhibits reuptake of dopamine, may influence food intake and body weight via the reward system.^66^

Subjective appetite is accessed by measuring desire to eat, hunger and prospective food consumption^19, 67^ using a visual analogue scale. Evidence shows that diet-induced weight loss in obese adults is accompanied by an increase in all components of appetite.^67^ Seventeen adults underwent a 33-week weight loss programme and were assessed using a visual analogue scale. There was an apparent increase in their fasting desire to eat, hunger and prospective food consumption.^67^ Moreover, a long-term study in 50 overweight or obese individuals showed that increases in the three components of appetite following a 10-week diet-induced weight loss programme remained elevated at ~1 year.^19^ Mean ratings of hunger, desire to eat and prospective consumption were significantly (*P*<0.001) greater at weeks 10 and 62 compared with baseline.^19^ There was a non-significant increase in ratings for preoccupation with thoughts of food at Week 10 (*P*=0.09), but a significant increase at week 62 (*P*=0.008).^19^

Furthermore, cravings are a component of the hedonic response to food. It has been shown that dieting or restrained eating generally increases the likelihood of food cravings,^68^ although, over the longer term, dieting actually reduces cravings for high-fat and carbohydrate-rich foods.^69^ Evidence indicates that the ability to mobilise neural circuits involved in executive control, particularly to resist food-related cravings, may be a component of successful outcome following gastric bypass surgery.^70^ In a functional magnetic resonance imaging study of 31 post-surgical patients, who were asked to view images of food, instructions to ‘crave' or ‘resist' elicited activity in the dorsomedial prefrontal cortex and dorsolateral prefrontal cortex, respectively.^70^ The more successful participants (that is, those meeting 50% excess weight loss) had the greatest activity in the dorsolateral prefrontal cortex when instructed to ‘resist'.^70^

Another functional magnetic resonance imaging study, investigating brain region-specific neural activity elicited by food-related visual cues in obese individuals, showed that changes in neural activity occurred in brain areas involved in the regulatory, emotional and cognitive control of food intake following stabilisation at a 10% reduced body weight.^71^ Among the changes during maintained weight loss, there was an increase in neural activity in systems relating to sensory responses to food and decreases in systems relating to cognitive control of food intake. Interestingly, many of the changes were reversed by injections of leptin.

### Translation to the clinic

Obesity was considered to be bad habits before the National Institutes of Health consensus conference of 1985 declared it a disease.^72^ Despite this designation, the perception of obesity did not begin to change significantly until the discovery of leptin in 1994, when it was demonstrated that obesity can be caused by the loss of a hormone and reversed by its replacement.^73^ Nevertheless, it was not until 2013 that the American Medical Association recognised obesity as ‘a disease requiring a range of medical interventions to advance obesity treatment and prevention'.^74^ Thus, obesity joins hypertension and other chronic diseases as another disease associated with serious health consequences requiring ongoing management. Accordingly, much can be learned from the development of treatments for hypertension, and development can be considered in the same three categories around which this paper has been organised: environmental, behavioural and homoeostatic.

From the environmental perspective, reductions in food intake, dietary modifications and environmental modifications (for example, walking-friendly cities) are logical interventions to address the obesogenic environment, since they are simple and safe. Unfortunately, dietary strategies—despite their inherent logic and appeal—have not been successful in making an impact on hypertension or obesity at the population level, and environmental modifications, such as changing city structures, can be expensive. The Dietary Approaches to Stop Hypertension diet was shown to be as effective as a single anti-hypertensive medication and is recommended as an initial intervention for hypertension, based on a clinical trial.^75^ However, in real life, patients with hypertension often find it difficult to adhere to a dietary regimen and to restrict their salt intake.^76, 77^ The prevalence of obesity began rising around 1980, despite recommendations to reduce caloric intake and increase physical activity. Thus, notwithstanding their inherent appeal, dietary modifications are difficult to successfully put into action because of the physiology associated with reward systems. A diet high in fibre and low in calories may be a logical recommendation, but people find it less appealing to eat than the foods they particularly like, which are high in fat and calorically dense.

Behavioural lifestyle modifications have been shown to help with weight loss^34^ and, to a certain extent, with hypertension, particularly over the short term.^78, 79^ Diseases that require ongoing management have physiological controls, and it is difficult to overcome physiology with behaviour over the long term. Because of the advances in electronic media, there are now more tools to help modify behaviour. Smartphone applications have been used for assessing food intake via food photography,^80^ and mathematical modelling^81^ may augment electronic systems and, thus, raise their success and cost-effectiveness in clinical practice settings, relative to traditional face-to-face counselling. These new technological advances could help improve behavioural therapy for obesity in the future, but they do not address the physiological controls that promote weight regain and, therefore, a healthy weight will still be difficult to maintain over the long term.

The homoeostatic perspective appears to hold the greatest promise in solving the problem of weight regain after weight loss. Unfortunately, despite the early potential of leptin to promote weight loss, results from clinical studies of exogenous leptin therapy have been variable.^82^ Leptin administration is unlikely to be effective in inducing weight loss as a stand-alone intervention, although it could potentially supplement or prolong the period of weight loss if administered with a ‘leptin-sensitising agent' that may overcome the effects of negative energy balance on leptin responsiveness.^82^ Leptin may still hold promise as a weight maintenance therapy in people who have previously undergone weight loss.^82^ Obesity surgery such as the gastric bypass, which modifies the physiology of body weight regulation, is clearly the most efficient treatment for obesity today.^83^

Treatments for chronic diseases tend to evolve through a series of steps, and the history of hypertensive treatment may be predictive of what to expect with obesity treatment. The first intervention in hypertension beyond diet was surgical sympathectomy, which was practiced in the 1940s.^84^ In the 1950s, thiazide diuretics appeared on the scene as a treatment for hypertension and caused the loss of sodium in the urine, which can be compared with orlistat, which was approved in the 1990s for obesity and caused fat loss in the stool. The next step in medications for hypertension acted on the brain, reserpine being one example.^85^ The brain is so proximal in the control system that unwanted side effects, for example, depression, can trickle down to other systems. Obesity drugs, such as amphetamine, can be viewed as comparable; except being associated with addiction and stimulation, rather than depression.^86^ The next step in anti-hypertensive medication development was combining medications in lower doses, in order to increase efficacy and reduce side effects. In the case of hypertension, the combination of hydralazine, reserpine and a diuretic is an early example.^85^ A parallel in the treatment of obesity is the combination of phentermine (a drug related to amphetamine) and topiramate extended-release, which was approved in 2012. The next step in anti-hypertensive drug development was treatments that acted on the target and had few side effects, due to their distal site of action in the system, for example, angiotensin receptor blockers, which act on the blood vessels. Obesity medicine is still at the stage where medications act on the brain or are combinations of drugs. Liraglutide 3.0 mg is a GLP-1 analogue and has recently been filed with regulatory authorities in the US and Europe for the treatment of obesity. By increasing GLP-1 signalling, liraglutide 3.0 mg acts directly to help address the downregulation of appetite-suppressing hormones observed after diet-induced weight loss.^87, 88^ Beloranib, a methionine aminopeptidase-2 inhibitor, is being investigated as an anti-obesity drug in Phase II clinical development. It appears to act peripherally, since it is effective even in the presence of obesity due to hypothalamic damage in rodents,^89^ and is also effective in Prader–Willi syndrome, a genetic type of human hypothalamic obesity with severe hyperphagia.^90^ Therefore, it appears that peripherally acting drugs for the treatment of obesity with a low risk of side effects are on the horizon and may represent the counterpart of what is now available for hypertension.

Thus, the environment and behaviour are important aspects of addressing the burgeoning obesity problem, as they continue to be in the field of hypertension, and technological innovations are likely to increase the cost-effectiveness of behavioural strategies. With similarities again to the experience with hypertension, homoeostatic and physiological approaches appear to offer the greatest hope for the prevention of weight regain, as a means of successfully addressing the obesity epidemic.

### Summary

Ensuring maintenance of weight loss is a crucial step in reversing the current and alarming rise in obesity and, hence, reducing the burden of obesity-related comorbidities. Restricting food intake through dieting generally leads to successful short-term weight loss, but, over the long term, many individuals regain the lost weight. Individuals who live in an ‘obesogenic' environment encounter opportunities to overeat on a regular basis. Moreover, compensatory physiological adaptations following diet-induced weight loss, such as decreases in energy expenditure, fat oxidation and anorexigenic hormone (for example, leptin) levels and increases in appetite, craving and orexigenic hormone (for example, ghrelin) levels, promote weight regain. There is also increasing evidence regarding the role of other factors, including hedonic factors and glial cell activity, in overriding the normal feedback loop controlling body weight.^13, 20, 25, 26, 28, 29^

In order to maintain a healthier weight, individuals must adhere to obesity-reducing behaviours that counteract physiological adaptations and other factors associated with weight regain. Creating an environment that favours healthy behaviour against the current backdrop, which encourages a positive energy balance has been, and is likely to remain, difficult.^15^ Although one size does not fit all in successfully maintaining weight loss,^91^ the use of anti-obesity medications and/or bariatric surgery are useful interventions that help to reset an individual's physiology. In the long term, interventions that alter the physiology of body weight control are likely to be more efficacious than behavioural interventions, such as counselling and social support to influence an individual's motivation and perseverance.^15^ The control of body weight physiology will allow adoption of lifestyle modifications, including eating a low-calorie diet, and undertaking increased physical activity.^15^

There is a continuing need to develop effective weight loss management strategies that, similar to anti-obesity medications, alter the physiology of body weight favouring weight loss and maintenance. Approaches that address the hedonic response to food or prevent chronic glial cell activation should also be explored as they may also be of benefit in preventing weight regain. Having an increased understanding of weight loss and the physiology of weight regain will facilitate the development of future strategies, including better methods of lifestyle modification (diet and exercise), but with emphasis on pharmacological and/or surgical interventions, to help post-obese individuals maintain their loss of body weight.

## Figures and Tables

**Table 1:** Environmental factors potentially influencing body weight

*Environmental factors*	*Overview*
Obesogenic environment	• Increased opportunity to over-consume large portions of energy-rich foods, coupled with a decline in physical activity, has an impact on energy homoeostasis
Infection	• Functional relationship between adipose tissue and the immune system,^32^ e.g., adipose tissue has a role in mediating inflammation
	• Certain immune cells, macrophages and adipocytes have similar functional characteristics, and pre-adipocytes can differentiate into macrophages;^92^ the potential for adipose tissue to expand in response to infection is feasible and result in a state of surplus energy^32^
	• The adenovirus 36 in the D group (AD-36) causes obesity in non-human primates,^93^ and humans with antibodies to AD-36 are more obese.^94^ In identical twins discordant for AD-36 antibodies, the positive twin is more obese, demonstrating the environment rather than genetic basis for the obesity^94^
Intrauterine and intergenerational effects	• The perinatal environment can influence the susceptibility of offspring to future metabolic challenge (metabolic programming)^95^
	• Children of obese mothers (or those who had excessive gestational weight gain) are at an increased risk of obesity because of *in utero* exposure to over-nutrition and associated developmental programming, and environmental exposure to a similar obesogenic lifestyle^95, 96, 97, 98^
Epigenetics^a^	• Epigenetic modifications represent a potential way in which ‘metabolic programming' can manifest^95^
	• Environmental factors during development can lead to permanent changes in epigenetic gene regulation,^99^ and epigenetic dysregulation may contribute to obesity^32, 95^
	• Transgenerational epigenetic regulation of metabolism and reward circuitry may influence the development and health of subsequent generations of offspring^16^
Increasing maternal age	• Mean pregnancy age has steadily increased and maternal age at time of birth has been correlated with different parameters by which obesity is defined^32, 97, 100^
	• A possible synergistic role of maternal comorbidities should be considered:^32^ older women potentially have more comorbidities, such as obesity, insulin resistance and hypertension, which may further increase the risk of obesity by the effect on the intrauterine environment. In addition, older women tend to have larger babies^32^ and larger babies have an increased risk of obesity later in life^96^
Sleep debt	• Circadian desynchrony is a characteristic of shift work and sleep disruption in humans, and implicated in metabolic pathologies^101^
	• Study findings indicate a relationship between the incidence of obesity with disrupted or decreased amount of sleep;^32, 101^ the association is observed across groups encompassing a range of ages and ethnicities, and has prompted initiation of mechanistic studies^32^
Iatrogenic effects of pharmacotherapies	• Weight gain is associated with a number of frequently used medications.^32^ In the case of psychotropic medications, such as anti-psychotic agents, there is substantive evidence to support weight gain in those receiving these drugs^32, 102, 103^
	• Despite challenges in estimating the full extent of drug-induced weight gain, the observation that some of the most commonly prescribed classes of drugs can lead to weight gain supports the view that drug-induced weight gain is contributing to the current obesity epidemic^32^

a Epigenetics refers to the post-translational modifications of DNA that result in differential levels of gene expression without altering the DNA sequence itself.
